# Supplementary material for: Content-rich biological network constructed by mining PubMed abstracts
Source: BMC Bioinformatics. 2004 Oct 8;5:147. doi: 10.1186/1471-2105-5-147 (PMC528731; doi:10.1186/1471-2105-5-147)
Supplement: Additional File 2 — The original results of the above study (non-essential files are deleted to keep the file size under the limit set by BMC bioinformatics). [file 1471-2105-5-147-S2.bz2 › chilibotAdditionalFile2/dip10/611ID10580496E10994/html/ATPE_ATPB.html]

 


 **ATPE** and **ATPB** 
  
Found 26 abstracts in PubMed, retrieved 10.  
 

 What does Google say? 
 PDF only 
| .edu only 

---

**Interactive relationship** (e.g. stimulation, inhibition, etc)

**Neutral relationship**- The 5 end of the  **atpE**  specific transcript has been mapped 430 431 nt upstream of the  **atpE**  translation initiation site, within the coding region of the  **atpB**  gene.  Ref: 7859310 Curr Genet, 1994

**Non-interactive relationship** (e.g. studied together, co-existance, homology, etc.)

- An  **atpE**  specific promoter within the coding region of the  **atpB**  gene in tobacco chloroplast DNA.  Ref: 7859310 Curr Genet, 1994
- The genes for the five subunits were present in the order atpI subunit I  [ **ATPB** ] ,  **atpB**  subunit a,  **atpE**  subunit c, atpX subunit b, and atpF subunit b.  Ref: 9818357 Arch Microbiol, 1998
- The  **atpB**  and  **atpE**  genes encode beta and epsilon subunits, respectively, of chloroplast ATP synthase and are co transcribed in the plant species so far studied.  Ref: 7859310 Curr Genet, 1994
- We estimate that the functional half life of  **atpB**  is slightly over one half that of  **atpE**  and the other atp genes, while atpI is at least two times less stable than  **atpB** .  Ref: 8679701 Biochim Biophys Acta, 1996
- No genes for photosynthetic functions have been found, except for sequences encoding six subunits of the ATP synthase atpA,  **atpB** ,  **atpE** , atpF, atpH, and atpI.  Ref: 12111556 Mol Genet Genomics, 2002
- The C. thermoaceticum atp operon contained nine genes arranged in the order atpI i,  **atpB**  a,  **atpE**  c, atpF b, atpH delta, atpA alpha, atpG gamma, atpD beta, and atpC epsilon.  Ref: 9171425 J Bacteriol, 1997
- Eight genes atpI,  **atpB** ,  **atpE**  1,  **atpE**  2,  **atpE**  3, atpF, atpH, and atpA upstream of and contiguous with the previously described genes atpG, atpD, and atpC were cloned from chromosomal DNA of Acetobacterium woodii.  Ref: 10567365 J Biol Chem, 1999
- Promoter leader regions of the  **atpB** ,  **atpE** , rps4, and rps7 genes representing the diversity of chloroplast SD like sequences were fused to aadA and uidA reporter genes encoding spectinomycin resistance and GUS activity respectively.  Ref: 9520261 Mol Gen Genet, 1998
- The ninth KpnI fragment about 4.0 kb of broad bean chloroplast DNA contains the entire chloroplast  **atpE**  gene and  **atpB**  gene.  Ref: 12168017 Sheng Wu Hua Xue Yu Sheng Wu Wu Li Xue Bao Shanghai < MedlineTA>Sheng Wu Hua Xue Yu Sheng Wu Wu Li Xue Bao Shanghai , 1998
- A nucleotide sequence of 7556 bp contained structures similar to those of ndhK, ndhC, trnV, trnM,  **atpE** ,  **atpB** , rbcL, trnR and accD.  Ref: 8604330 Nucleic Acids Res, 1996
- The genes were organized in an operon with the gene order atpEBFHAGDC ; i.e., the order of  **atpE**  and  **atpB**  is reversed with respect to the more typical bacterial organization.  Ref: 10940012 J Bacteriol, 2000
